# Editorial: Spatiotemporal regulation of central nervous system disorders: molecular mechanisms and emerging techniques

**DOI:** 10.3389/fcell.2023.1301013

**Published:** 2023-10-06

**Authors:** Yanrong Zheng, Weiwei Hu

**Affiliations:** ^1^ Key Laboratory of Neuropharmacology and Translational Medicine of Zhejiang Province, College of Pharmaceutical Sciences, Zhejiang Chinese Medical University, Hangzhou, China; ^2^ NHC and CAMS Key Laboratory of Medical Neurobiology, Department of Pharmacology and Department of Pharmacy of the Second Affiliated Hospital, School of Basic Medical Sciences, College of Pharmaceutical Sciences, Zhejiang University, Hangzhou, China

**Keywords:** CNS disorders, spatiotemporal regulation, cell subpopulation, cell type-specific, neurodevelopment, spatially resolved transcriptomics

Biological events occurring in the human brain are elegantly tuned to impart diverse emotions and elaborate behavioral patterns. The role of signaling pathways often changes during the progression of CNS disorders, and there is remarkable spatial heterogeneity in cell types and molecular expression profiles over the brain. Therefore, a full temporal and spatial illustration of the pathological progression of CNS disorders would benefit the development of novel therapeutic strategies. In this Research Topic, 6 articles, including high-quality original research articles and comprehensive reviews, discuss the spatiotemporal regulation patterns of CNS disorders and emerging techniques in this field.

Temporal lobe epilepsy (TLE) is the most prevalent focal seizure, which is highly likely to progress to generalized seizures. Here, Zou et al. explored the cell type-specific modulatory roles of dopamine receptor D1 (D1R)- and D2R-expressing medium spiny neurons in the nucleus accumbens shell in TLE. They found that cell-type-specific inhibition of either D1R-MSN or D2R-MSN alleviated TLE seizures by reducing the number of secondarily generalized seizures and improving seizure stages, without altering the onset time of focal seizures. The data of the aforementioned study along with other studies suggest that different brain areas and cell populations participate in seizure initiation, propagation and termination, thereby highlighting the necessity of demonstrating the spatial heterogeneity of pathological mechanisms of epilepsy.

Similarly, Zhou and Zhang. discussed the cell type-specific role of oligodendrocytes in various CNS disorders and delineated the contribution of myelin to neuronal activity. Interestingly, neuronal excitability can also affect myelin functions. The authors overviewed the potential strategies for myelin regeneration through the precise modulation of neuronal activity.

In addition to understanding the functional heterogeneity of different cells and brain areas spatially, the illustration of temporal pathological mechanism of CNS disorders is relevant to future whole-course disease management. In this context, Yi et al. reviewed the temporal regulation of key biological events that occur during neurodevelopment after experiencing febrile seizures. Febrile seizures are quite common during early childhood (-4%) (Patel et al., 2015; Offringa et al., 2017) and recent clinical research has revealed a close association of febrile seizures with epilepsy and other neuropsychiatric diseases in adulthood. Therefore, analyzing the long-term outcomes of febrile seizures and deciphering the underlying mechanisms may considerably contribute to the treatment and prevention of neurological and psychiatric disorders.

The aforementioned studies dissect pathological mechanisms of CNS disorders from a spatiotemporal perspective. Similar research approaches can also be used to study the pharmacological mechanisms of drugs and other functional compounds. Lai et al. examined the molecular mechanisms involved in the treatment of chronic pain using sinomenine, an active ingredient in the natural plant ‘sinomenium acutum (Thunb.) Rehd. Et Wils’, while focusing on its regulatory roles in distinct immune cell subpopulations. Sinomenine can regulate the interaction among different immune cells, immune cells and neurons and glial cells and neurons to confer immunosuppressive effects.

Studies focusing on the spatiotemporal pathological mechanism of CNS disorders will benefit from the newly developed techniques providing spatiotemporal information. Wong et al. proposed a novel electrochemical localized oxygen scavenging system (eLOS) to induce hypoxia *in vitro* with spatiotemporal precision. Focal hypoxia is very common in multiple diseases such as ischemic stroke, cardiac arrest and dementia; however, the currently available experimental model of spatially restricted ischemia is limited. Using the eLOS platform, Wong et al. successfully induced axon-restricted hypoxic stress in human cortical neurons and found that localized axonal hypoxic stress induced neuronal death even when the somas remain in the normoxic culturing condition. These findings indicate the potential of the eLOS platform in studying cellular and subcellular changes in white matter after an injury and other hypoxic insults.

Furthermore, Ya et al. illustrated working principles and provided examples of the application of cutting-edge spatially resolved transcriptomic technologies in studying CNS disorders. The rapid development of these technologies will eventually provide a deeper understanding of the molecular mechanisms underlying CNS disorders.

Overall, it has become inevitable to elucidate the pathological progression of CNS in high spatiotemporal resolution. We hope that this Research Topic will help readers understand CNS disorders from a highly spatiotemporally precise perspective.
